# Illinois State Dental Society

**Published:** 1888-09

**Authors:** C. N. Johnson


### ILLINOIS STATE DENTAL SOCIETY.

TWENTY-FOURTH ANNUAL MEETING.

REPORTED FOR THE INDEPENDENT PRACTITIONER.

BY C. N. JOHNSON, L. D. S., D. D. S.

CONTINUED FROM PAGE 423.

THURSDAY EVENING SESSION.

A paper by Dr. L. P. Haskell was then read: "Prosthetic Dentistry—Some Difficult Cases and their Treatment." He said that if the conditions of all mouths were similar and equally favorable, the insertion of dentures would be a pastime to the experienced dentist. It is not so, and the unfortunate thing is that patients will not recognize this. First study all unfavorable conditions, for no matter how long we practice, new combinations will present themselves. If the case is difficult, first impress the patient to this effect; control the patient, or you cannot properly control the mouth.

In cases where all the upper teeth are out, and all the lower except the anterior ones, the patient cannot successfully wear the upper plate without lower back ones, as the constant pressure in front will cause absorption of the process, and leave a yielding ridge of gum in the anterior portion of the upper jaw.

Where, in these cases, one or two bicuspids remain on one side in the lower jaw and not on the other, remove them to get even pressure. Again, where all the upper teeth are out except the posterior ones on one side, they had better be removed, but sometimes we may clasp, and use a suction plate, leaving the teeth in.

Continuous gum is the only material where the upper jaw is prominent and the lips short.

Where all the upper teeth and the anterior ones of the lower jaw remain to gain masticating surface, put in a lower posterior set, but do not raise them high to relieve the pressure from the anterior ones, as the gums will yield and cause discomfort. In these cases it is preferable to "shoe" the front ones.

The most important thing in artificial work is the articulation.

In this connection observe three rules: Never allow pressure on the six anterior teeth; never in full upper plates have the pressure greater on one side than the other; never allow the second or third lower molar, which has projected forward so that its face shows, to meet an artificial tooth at that angle, as it will crowd the upper plate forward.

As a rule, a full lower plate is more useful and comfortable than a partial, because the pressure is equally distributed over the whole jaw.

DISCUSSION.

*Dr. E. D. Swain*—This is an important question to us as a profession. It would seem as though it had been tabooed of late. Nothing new has been given us for years, except artificial crown and bridge-work. It has been neglected in our schools. Students are not properly instructed on this subject, and much that is erroneous is taught them. In regard to difficult cases and how to overcome them, we should first consider the diagnosis of the case, and study it well. Get a correct impression in plaster of paris. The most difficult cases in which to get a perfect impression are those having uneven surfaces, soft in one place, hard in another. In such cases scraping the model is not efficient; few men have nicety of judgment sufficient to do this accurately. I take an impression in wax, then trim it out and use it for a cup, in which I take a second impression in plaster.

Other difficult cases are those in which the alveolar process is absorbed in front, especially in the upper jaw. The ridge is loose and flabby, and in taking the impression it is pushed out toward the lip. In these cases cut the forward projection from the model, and add wax to the ridge to reproduce the natural form of the mouth.

Lower partial plates are usually discouraged by dentists, and it is true that they are sometimes very troublesome in our hands. A rule that I have followed with success in partial and full lower plates, is to cut away till I feel sure I have destroyed it, and then cut away some more. I do not hesitate to use clasps in these cases, even if I know the tooth will be ruined in four or five years. There is more benefit to the patient from the successful use of the plate than from the tooth clasped.

The essayist placed stress upon the excellency of continuous gum-work. There is no question that it is the cleanest and best material, with the possible exception of all-porcelain. Continuous gum is sometimes too heavy, bungling and clumsy, and the dentist must discriminate as to the material. Rubber has been of much value, and cast metal plates have their place, so that the observing dentist should know which to use.

As to the question of extracting teeth, to render an artificial denture a success, it is sometimes advisable to extract perfectly sound teeth, if by so doing we can benefit the patient.

The paper laid particular stress on the importance of proper articulation in dental substitutes, and the three rules laid down are universal.

The discerning dentist who travels, or who walks on the streets, is hurt by the artificial teeth he meets, from the manner in which they are put together. They are seldom one-third as large as they ought to be, and often point into the mouth, showing all the teeth when the mouth is open. There is no effort to build out the teeth and restore the features. Mechanically, they are all right, for if the blow comes inside the arch the tendency is to hold the plate up firmly, and they are worn with comfort. As to the satisfaction given by artificial teeth, it is safe to say that a perfectly satisfactory set of teeth cannot be made.

*Dr. Taylor*—There is one difficult case not spoken of; where all the teeth back of the cuspids are absent in the upper jaw. The air gets under the plate in front, and it is exceedingly difficult to keep it up. In one very stubborn case of this kind I made a deep air-chamber, and it worked satisfactorily. It is sometimes necessary to drop out two or three teeth to make them natural in appearance, using twelve instead of fourteen.

*Dr. Ames*—I do not advocate the extraction of sound teeth. I believe it is unjustifiable. It might be necessary in rare instances, as in a partial case where the space is not right, and in the upper jaw where only one or two teeth remain. In the latter case we might want to shut out the air and enclose the whole ridge, but these are the only cases. I would not extract sound teeth on one side to equalize the pressure, as the upper plate can be so constructed that this is not necessary. Generally, dentists do not run an upper plate up far enough over the tuberosity and out toward the malar process ; and they do not arrange the teeth so that the pressure is directed toward the center of the mouth.

Another thing, where an upper plate is necessary and any roots are remaining, the essayist would extract. I would fill them and place the plate over them. They will sustain the pressure, and the plate will not settle. In partial lower plates let a lug run up on the grinding surface of the natural teeth so as to let the force of mastication come on them. In the case mentioned by Dr. Taylor, I would depend more on the articulation than on an air-chamber. Get a forward pressure on the plate and it will be held in place.

Subject passed. Adjourned.

FRIDAY MORNING.

The first in order was a paper by Dr. J. Frank Marriner, on "Making and Tempering Instruments." He said :

"This paper is not intended for experts, but for those who are still in the dark on this subject. The making and tempering of dental instruments seems to be considered by many an exceedingly difficult and mysterious process; but the facts are that it is very simple, and easy of accomplishment by the average dentist.

"One of Fletcher's gas or gasoline forges, No. 19, will be found useful for this purpose, especially in small forging and repairing. Instructions for its use may be found in Fletcher's catalogue of 'Laboratory Apparatus.' It costs, with foot-blower, blow-pipe, and rubber tubing complete, about eighteen dollars. This, with a medium sized anvil and several hammers of different sizes, make a respectable outfit. If a man has a decided taste for this kind of work he may procure other appliances that will be of value, but for the ordinary dentist who wishes to make a new instrument only occasionally, this is an unnecessary expense, as he can accomplish his purpose with one of Fletcher's solid flame burners, No. 46, costing two dollars. Use with this a good mouth blow-pipe, and it will prove very efficient. In forging, the first thing is to have in your mind a perfect conception of the instrument you wish to make. Exercise care in heating, as steel is injured by too much heat. Do not use the hammer before the requisite temperature is obtained, and distribute the force as evenly as possible; that is, turn the steel at every blow, striking first on one side, then on the other. Always keep vividly in the mind's eye the form of instrument you wish to make, and conform every blow to this idea.

"Of course, in making small instruments, as thin scalers, excavators, etc., the forged instrument will be two or three sizes larger than needed when finished. Be sure and leave it large enough for grinding, but endeavor to be as exact as posssble in regard to the form. Hammer it into shape rather than use the file, as the instrument will be tougher, more springy, and have a better cutting quality.

"When the instrument is formed, use fine sand paper or emery cloth to remove the effects of the heat. This must be repeated at every subsequent heating. It is now ready for tempering. Use the solid flame burner with the blow-pipe. Gently rotate the instrument in the flame till it reaches a bright cherry red, then quickly plunge it into cold water. If you are in doubt as to the temper, test it with a file. If it is too soft, clean it as before and repeat the process, carefully observing the heat for a higher temperature. The beginner may have to repeat several times.

"With the instrument highly tempered, go over it again, using sand paper, etc., to remove the effects of the heat, being careful not to break it, as it is, in its present state, capable of little resistance.

"The next step is to draw the temper, so that we may have a spring at the neck and shank, leaving sufficient hardness at the cutting edge. To do this, force the blade into a piece of soft pine about an inch square, and throw a flame from the blow-pipe upon the larger part of the shank till it assumes a blue color. As the color passes up the neck, if the point in the wood be small, stop it just before it reaches the wood; but if large, let the color run to the wood, and then check it instantly. This will give a strong shank, spring temper at the neck, and keen cutting edge that will not crumble or turn in cutting tooth substance.

"In tempering long bladed instruments, as thin scalers, excavators, etc., do not force more than one-third or one-half of the blade into the wood, and as the color passes over the neck and down the blade arrest it in a bright straw color just before it reaches the wood. This gives a spring temper throughout.

"Now comes the important point of grinding the instrument. Keep the stone wet and place the instrument on the stone in precisely the position that you wish it to remain throughout the grinding. On removing for any cause, be particular to return it in the identical position it occupied before. In this way you can grind a delicate instrument accurately; and delicacy is of the utmost importance. Not one dental instrument in ten is made small enough. They are clumsy, and they are not tempered properly. They are too rigid. We want instruments that will cut tooth substance, and not batter it down by force. As an example in fine instruments, compare Dr. Cushing's scalers with those usually found on the market, and when you have used the Cushing scalers once, you will wonder how you ever succeeded with others."

DISCUSSION.

*Dr. Cushing*—There is hardly any discussion necessary, as the paper covered the ground completely, and gave good instructions. I might say a word or two as to the importance of the dentist being able to make his own instruments. This accomplishment is more valuable to the country dentist, who is removed from the depots. Many times an instrument will break, and if he has only one of that kind, he is often obliged to do his work with one not adapted to the purpose. If he could make his own instruments he would not be caught in this dilemma, as one who understands well how to make an instrument can do so in five minutes.

Most dentists who are in the habit of making over instruments usually make poor ones because they have not studied the nature of steel. There is not one in ten, of those who handle steel, who is thoroughly skilled in regard to tempering. The manufacturers all fail to give us the kind of instruments we need. I seldom buy hatchets or small excavators but that I have to temper them; they may be of good shape, but are not well tempered. As an illustration of the necessity of understanding the metal upon which we are working, I remember when in California many years ago, in the mines, we had to have our picks sharpened often, and we invariably went to a blacksmith ten miles distant, in preference to those living nearer, because he always sharpened them just right. He understood his business, and they did not. He studied how to manage steel. There are no two lots of steel exactly alike, and each lot must be manipulated differently. All the steel in one lot is alike, and we must learn by experience how to treat that lot. This was the secret of the blacksmith's success.

The principal points in making instruments are : First, do not overheat; the instrument may easily be spoiled by this. It should not be carried beyond a cherry red in forging. Never go to a white heat ; it is better to reheat often rather than heat too much. Keep turning the instrument when forging so as to render the steel compact. Do not spread it out too much or it will not be homogeneous. In tempering, heat to the lowest possible degree that will harden the steel. Usually this will be a low cherry red, but sometimes you will have to carry it to a brighter red. Then, for drawing the temper place the end of the instrument in a piece of wood, and heat till the blue runs up to this. Grind your instrument down, and you will then have a better one than you can buy.

I would emphasize the necessity for having more delicate instruments. The ordinary hatchet excavators are too clumsy. Better work can be done with greater comfort to the patient by the use of thin instruments, and they will last well if tempered in this way. Do not expect one of these instruments will stand what a large pick will; they are not for breaking down, but for cutting tissue.

One word of advice—have a good sized whetstone, and use it often. With dull instruments you only rasp the tissue off. Small stones are useless, as a groove is soon made in the centre. Use a broad stone where you can sweep across a large area. Arkansas stone is the kind to use. The point is to resharpen often. I have been annoyed very much in the clinic room at the college to note the indifference of students in this regard; and the majority of practitioners have the same careless habit. Use keenly sharp instruments.

*Dr. Ames*—I would like to ask Dr. Cushing what the color of the point is in drawing the temper?

*Dr. Cushing*—The same as before; you do not wish to draw the temper at the point.

*Dr. Ames*—Then a good article of steel is not brittle when hardened?

*Dr. Cushing*—If you heat just to the point of hardness, and not beyond, you get hardness without brittleness.

*Dr. Morrison*—I have been much interested and benefited by the paper. I am astonished at the condition of the instruments in many offices. When an instrument breaks it is sometimes a good thing, as a better one can be made on the handle.

I would make one issue with the paper, that the instrument should be shaped more with the hammer than with the stone. The advantage is that the very compression you have made will leave the outside shell a little harder than the rest. Now, in shaping the blade, forge down to a sharp edge; sharpen it with the hammer. Use absolutely cold water in hardening. To draw the temper, instead of wood use tin or lead. Sink the end in lead and carry it over a Bunsen burner. You can watch it better than with the blow-pipe. Give a straw color just where it enters the lead.

Regarding drills for retaining pits or removing fillings, when a small bur breaks, shape it, temper it, and use it for these purposes.

*Dr. Cormany*—I should advocate the use of soap in tempering. You will get better results by its use than by any other method.

*Dr. Taggart*—There is a little work published by the Crescent Steel Co., Chicago, on the manipulation of steel from a scientific standpoint. It is for free distribution, and is a valuable work, well worth reading and studying.

Steel should be worked properly from the beginning. When you break an excavator and have difficulty in tempering it, the fault may be in the original working.

Yellow soap with rosin is best for tempering. Smear it over and the scale will not form ; you will have a smooth surface. If you want a really hard instrument, plunge it into mercury.

*Dr. Wassall*—There is one method of heating not mentioned, that is, in superheated lead. To temper Swiss broaches, place them in one of Williams' cylinder bottles, with an opening in the cork so the bottle will not burst. Then heat gradually, and you can watch the color of the broaches. I would ask the essayist what kind of a grindstone he uses ?

*Dr. Marriner*—Corundum.

*Dr. Cushing*—In reply to Dr. Morrison, I think it is a fallacy not to grind the instrument after heating. I have been informed by a gentleman who understands perfectly the working of steel, that this is one of the most important points.

*Dr. Ames*—Since Dr. Taggart has given us such an admirable machine for making corundum points for the engine, it will be found more advantageous to use these in grinding instruments, where fine work is required.

*Dr. Crouse*—Mr. President, I don't wish to make a speech on the subject before us, but I do want to compliment this body on the work they have done at this meeting. It does my soul good to see the members of this society earnest in trying to help each other and the better to fit themselves to practice dentistry.

To know how to practice dentistry in the most perfect manner certainly is a laudable ambition, and that seems to be the aim of these meetings. If the community depending upon us for good service could understand what real honest work this society is doing, they would surely rejoice, and our recognition would be quite as great as though we had spent the time in trying to find out whether or not we are medical men, or whether our standing in the community is as high as it should be.

Subject passed.

Dr. Patrick then made some impromptu remarks on "The Rationale of Constructing and Attaching Artificial Crowns to Natural Roots of Teeth."

He said: "When we attempt to fit a band to a root, we are dealing with the base of a cone, and the farther we extend the band on the root the worse off we are. We make it more insecure because the upper end of the band is so much farther away from the root, on account of the latter being broader at the end.

"The point is to make a band that will go down as far as possible without leaving a space. To do this, bevel the root a little on one side, fit the band tight, and slip it over the other side, bringing it into position on the root with a stretch. In this way a band can be placed on a root so firm as to render it impossible to pull it off by applying the force in a direct line with the root.

"Much has been said about posts. All roots have canals following more or less the external form of the root, so that you have the same principle involved in this. The farther you insert a square butted post the weaker you make the root. Where there is plenty of material it makes little difference, but ordinarily the post should be made tapering.

"It is, however, seldom necessary to use posts where the bands are perfect. Many gentlemen use binding wire in measuring the root for the band. Now, the more you twist that wire the farther down it goes, so you do not get a correct measurement, and moreover, you are using a round piece of metal in measuring, when you eventually put on a flat piece. It is always better to fit the gold itself. In placing a band on an incisor, first put it over the lingual surface and force it toward and over the labial, as the pressure on the crown will be in that direction."

He concluded by saying that the roots of teeth ordinarily remain healthy a much longer time than the crowns, and this encourages us to a constant study of the best methods of crowning.

